# Supplementary material for: Dermatological changes in a prospective cohort of acutely ill, hospitalised Malawian children, stratified according to nutritional status
Source: BMJ Paediatr Open. 2024 Jun 8;8(1):e002289. doi: 10.1136/bmjpo-2023-002289 (PMC11163641; doi:10.1136/bmjpo-2023-002289)

SCORDoK
Standardised clinical score of skin manifestation in SAM

ID:_______ **Phase:** Admission☐ Transition☐ Discharge☐

Body surface area

Skin manifestation	No	Yes	Grade I	Grade II	Grade III
Telogenic effluvium					
Pigmentary changes					
Ichthyosiform skin change					
Lichenoid skin change					
Bullae - Erosions- Desquamations					

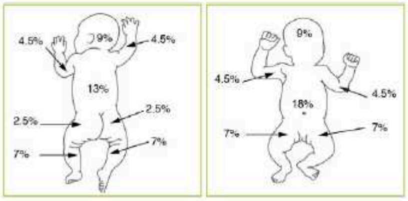

Supplement: Supplementary data [file bmjpo-2023-002289supp001.pdf]
