# Supplementary material for: Dermatological changes in a prospective cohort of acutely ill, hospitalised Malawian children, stratified according to nutritional status
Source: BMJ Paediatr Open. 2024 Jun 8;8(1):e002289. doi: 10.1136/bmjpo-2023-002289 (PMC11163641; doi:10.1136/bmjpo-2023-002289)
Supplement: Supplementary data [file bmjpo-2023-002289supp002.pdf]

SkinCHAIN Enrolment CRF v1
SkinCHAIN Number [3][0] [0][0][1] [ ][ ][ ]

Eligibility Checklist		
• Enrolled into the CHAIN study (Indicate Chain number:)	Y	N - ineligible
• Willingness to have a skin examination within 24 hours of admission and to be followed up at 6 months.	Y	N- ineligible
• Willingness to have photograph taken	Y	N- ineligible

Part 1

Admission to Hospital and SkinCHANGES Study Enrolment					
**DATE arrived at the hospital**	__ __/__ __/__ __ __ __ D D/M M/ Y Y Y Y				
**DATE of enrolment** i.e. date consented and seen by research team	__ __/__ __/__ __ __ __ D D/M M/ Y Y Y Y			**Sex**	☐ Male ☐ Female
**DOB**	__ __/__ __/__ __ __ __ D D/M M/ Y Y Y Y	**Is the DOB:**	☐ True ☐ Estimated*	**Child's Initials**	___ ___ ___

CURRENT HEALTH	
**Previously admitted to hospital.** *Include other hospitals / health centres. Select 1*	☐ No ☐ < 1 week ago ☐ 1 weeks-1month ago ☐ >1month ago
**Any medication last 7 days**. *Select all that apply*	☐ No medication ☐ Antibiotic ☐ Antimalarial ☐ Traditional ☐ Deworming ☐ Vitamin ☐ Yes, but unknown ☐ Other

SHORT SUMMARY OF PRESENT ADMISSION (Skip if not in-patient)

HIV STATUS: R/NR/UNKNOWN

PRESENTING COMPLAINTS AND DURATION:

……………………………………………………………………………………………………
……………………………………………………………………………………………………
……………………………………………………………………………………………………
……………………………………………………………………………………………………
……………………………………………………………………………………………………
……………………………………………………………………………………………………
……………………………………………………………………………………………

WORKING DIAGNOSIS:

DERMATOLOGICAL HISTORY		
Known chronic dermatological disease	Atopic eczema<br>Ichthyosis<br>Epidemolytic bulllosa<br>Other	Yes/ No<br>Y/N<br>Y/N<br>Y/N
Skin complaints before admission (tick all that apply)<br>[duration in weeks if known]	☐ Rash<br>☐ Itch<br>☐ Ulcers<br>☐ Swelling<br>☐ Other (specify)	[ ]<br>[ ]<br>[ ]<br>[ ]<br>[ ]

Examination	
Examination should be performed by the dermatologist who must formulate a diagnosis based on clinical history and findings.	
**Lichenoid skin changes**<br>*(select one)*	☐ **Grade 1** ☐ Grade 2 ☐ Grade 3<br>None
**Ichthyosiform skin changes**<br>*(Select 1)*	☐ **Grade 1** ☐ Grade 2 ☐ Grade 3<br>None
**Bullous erosions desquamation**	None<br>☐ Grade 1 ☐ Grade 2 ☐ Grade 3
**Telogen effluvium**	☐ **Yes** ☐ No Comments . . . .
**Pigmentary Changes**	☐ Y ☐ N Comment

CHAIN Enrolment CRF v1.61

CHAIN Number [3][0] [0][0][1] [ ][ ][ ]

General dermatoses diagnoses

Clinical diagnosis should be based on examination and investigation findings.

Tick the three most likely diagnoses.

**Bacterial infections**	**Infestations/Fungal Infections**	**Inflammatory disorders**
	☐ Tinea capitis	☐ Urticaria
☐ impetigo	☐ Tinea faciei	☐ Angioedema
☐ ecthyma	☐ Tinea corporis	☐ Papular pruritic eruption
☐ Erysipelas	☐ Scabies	☐ Papular urticaria/insect bites
☐ Cellulitis	☐ Creeping eruption	☐ Drug reactions
☐ Folliculitis		☐ Other
**Viral infections**	**Eczemas**	
☐ Molluscum contagiosum	☐ Atopic/Infantile eczema	**Fungal infections**
☐ Common warts	☐ contact eczema	☐ Tinea capitis
☐Flat warts	☐ Seborrhoeic eczema	☐ Tinea faciei
☐ varicella		☐ Tinea corporis
☐ Herpes zoster	☐ Other eczemas not classifiable	
		Others
		Tumours: KS
☐ Herpes simplex		Genodermatoses:
☐ Measles		Albinism, XP
	Papulosquamous disorders	Miliaria
	☐☐Psoriasis	
	Pigment disorders	
	Vitiligo	

GRADING OF SKIN CHANGES ACCORDING TO HEISLOV et al, 2015	
**DERMATOSIS**	**GRADING**
Telogen Effluvium	Yes (if present) and No (if absent)
Pigmentary Changes	Yes (if present) and No (if absent)
Ichthyosiform skin changes	**Grade 1:** Dry hyperpigmented skin with prominent skin lines and grayish scale<br>**Grade 2**: Hyperkeratotic areas and thick grayish scales<br>**Grade 3:** Hard to shiny hyperpigmented thick scaling which leave erosions on detachment
Lichenoid skin changes	**Grade 1:** 1 to 5mm hyperpigmented to purple brown flat papules<br>**Grade 2:** variable sized hyperpigmented, hyperkeratotic and lichenified well defined plaques<br>**Grade 3:** Thicker infiltrated plaques which may be shiny. May detach, leaving erosions or intact epidermis beneath
Bullae-erosion-desquamation	**Grade 1:** less than 5 % body surface area involvement<br>**Grade 2:** 5 to 30 % body surface area involvement<br>**Grade 3:** greater than 30% body surface area involvement
