# Supplementary material for: Dermatological changes in a prospective cohort of acutely ill, hospitalised Malawian children, stratified according to nutritional status
Source: BMJ Paediatr Open. 2024 Jun 8;8(1):e002289. doi: 10.1136/bmjpo-2023-002289 (PMC11163641; doi:10.1136/bmjpo-2023-002289)
Supplement: Supplementary data [file bmjpo-2023-002289supp003.pdf]

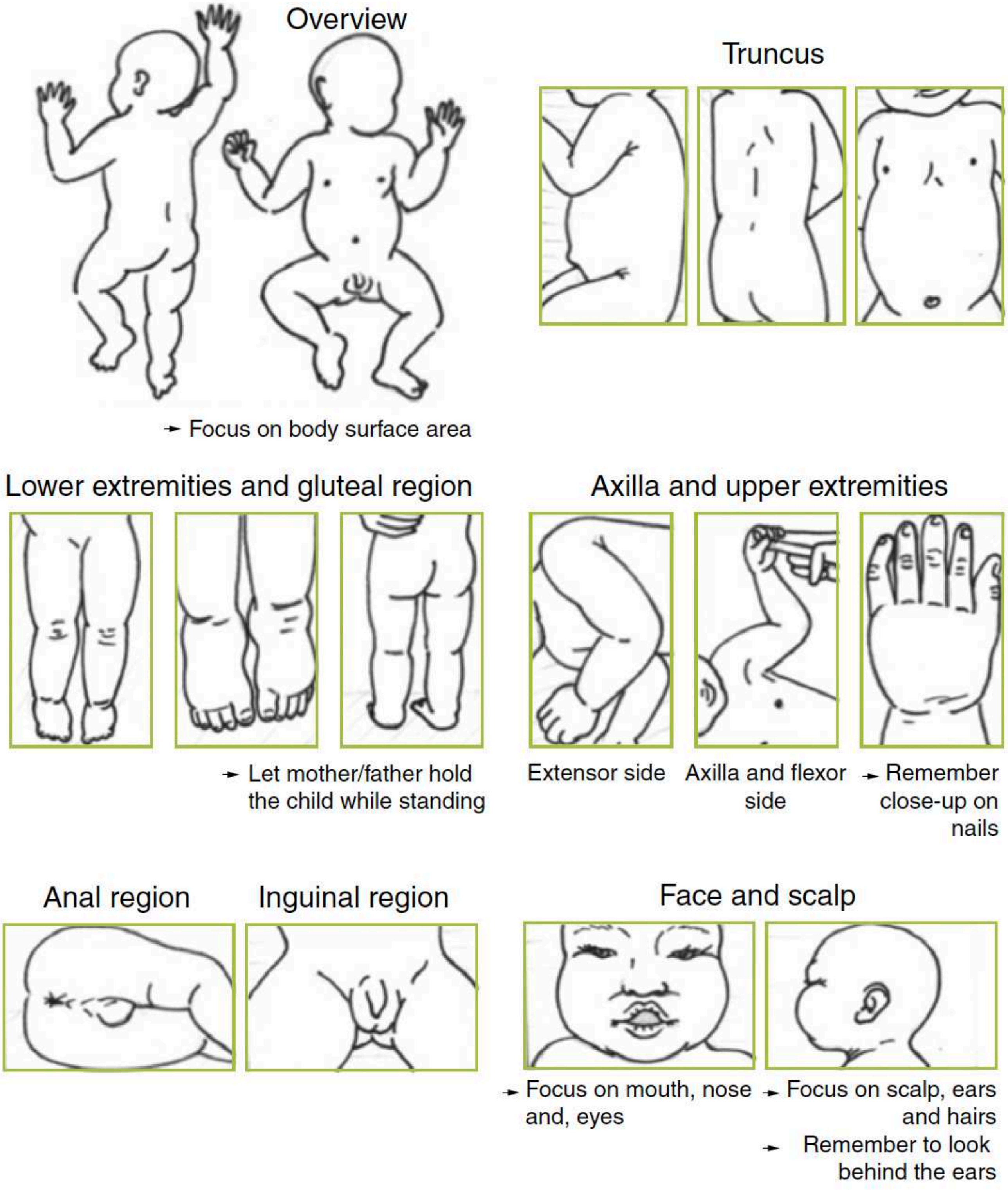
Overview
Truncus
Focus on body surface area
Lower extremities and gluteal region
Axilla and upper extremities
Let mother/father hold the child while standing
Extensor side
Axilla and flexor side
Remember close-up on nails
Anal region
Inguinal region
Face and scalp
Focus on mouth, nose and, eyes
Focus on scalp, ears and hairs
Remember to look behind the ears
